# Supplementary material for: Dermal macrophages set pain sensitivity by modulating the amount of tissue NGF through an SNX25–Nrf2 pathway
Source: Nat Immunol. 2023 Jan 26;24(3):439–51. doi: 10.1038/s41590-022-01418-5 (PMC9977679; doi:10.1038/s41590-022-01418-5)
Supplement: Supplementary file 1 — Supporting data for figures [file 41590_2022_1418_MOESM1_ESM.pdf]

nature immunology

Article https://doi.org/10.1038/s41590-022-01418-5

Dermal macrophages set pain sensitivity by modulating the amount of tissue NGF through an SNX25–Nrf2 pathway

In the format provided by the authors and unedited

Supporting Data for Fig. 3

e

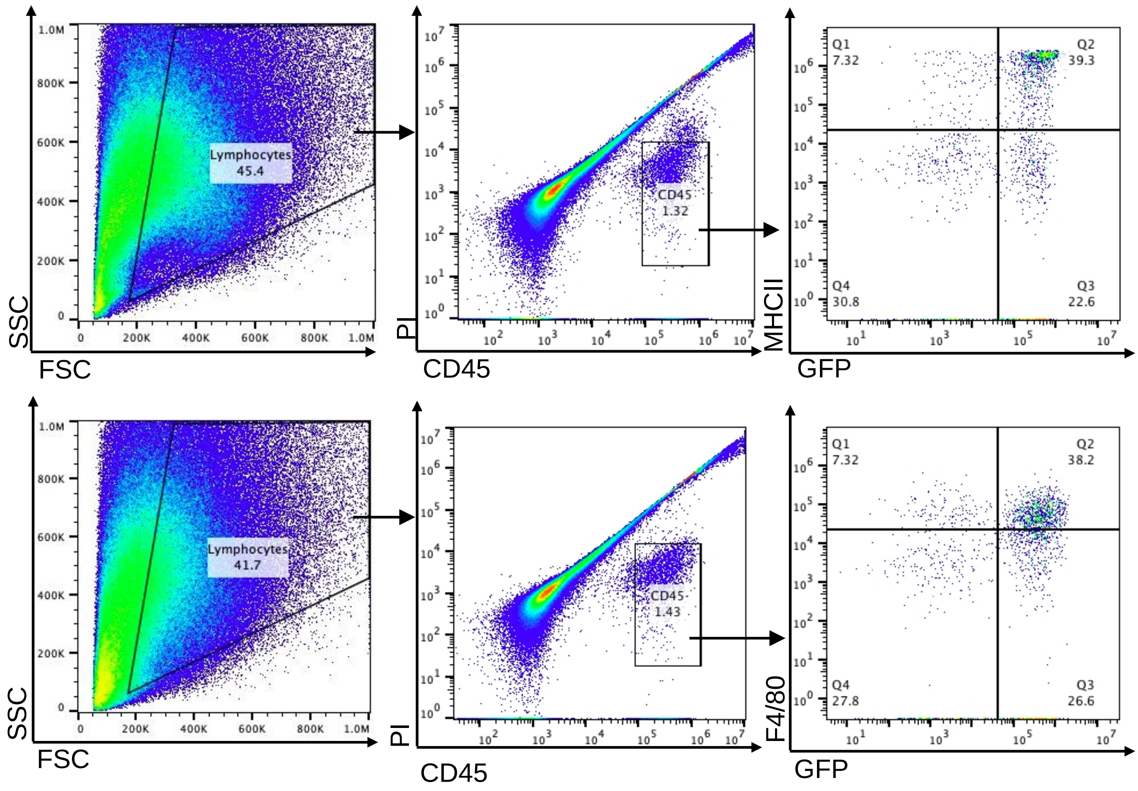

For the analysis of myeloid population in the skin, cells were obtained from hind paw using a Multi Tissue Dissociation Kit 1 (130-110-201, Miltenyi Biotec) with gentleMACS dissociator (Miltenyi Biotec) according to manufacturer's instruction. The hind paw skin were minced by razor blade, and then subjected to enzymatic digestion at 37 ^{o}C, for 2 h in rotation. During the enzymatic digestion, cells were dispersed by the programs (h_tumor_01, h_tumor_02, and h_tumor_03) of gentleMACS. Debris was removed by a 70-µm cell strainer. In some experiments, cells were separated with 30/70% percoll (17-0891, GE Healthcare) by the centrifugation for 20 min at 400 *g*. The cells were stained with various combinations of mAbs. FcγII/III receptors were blocked by prior incubation with anti-CD16/32 antibody (1:100, 156604, BioLegend). The mAbs used in this study were PE–anti-CD45 (1:100, 103106, BioLegend), Biotin-anti-MHCII (1:100, 107603, BioLegend), Biotin-anti-F4/80 (1:100, 123105, BioLegend) and second Ab APC-Streptavidin (1:200, 405207, BioLegend). To exclude dead cells from analysis, cells were stained with propidium iodide (PI, 421301, BioLegend). Cells were analyzed and sorted using Cell Sorter SH800S (Sony). Data were processed with FlowJo (v10, Tree Star).

Supporting Data for Fig. 3 (*Continued*)

g

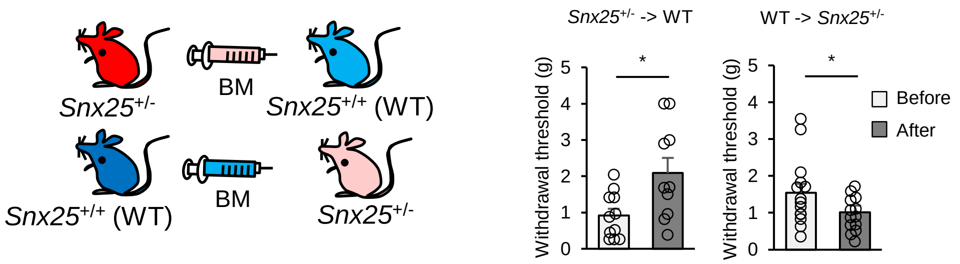

BM recipients were male 8-week-old *Snx25*$^{+/+}$, *Snx25*$^{+/-}$ or *Snx25*$^{fl/fl}$ mice. Mice were intraperitoneally injected with the chemotherapeutic agent busulfan (30 μg/g body weight; B2635, Sigma-Aldrich) in a 1:4 solution of dimethyl sulfoxide and PBS at 7, 5, and 3 days prior to bone marrow transfer. All mice were treated with antibiotics (trimethoprim (35039, Nacalai Tesque) and sulfamethoxazole (S7507, Sigma)) for 14 days after busulfan treatment. BM-derived cells were obtained from the femur and tibia of 5-week-old *Snx25*$^{+/+}$ mice, *Snx25*$^{+/-}$ mice and resuspended in PBS with 2% FBS. BM-derived cells (1×10^6) were transferred to recipient mice by tail vein injection.

Supporting Data for Fig. 4

h, i

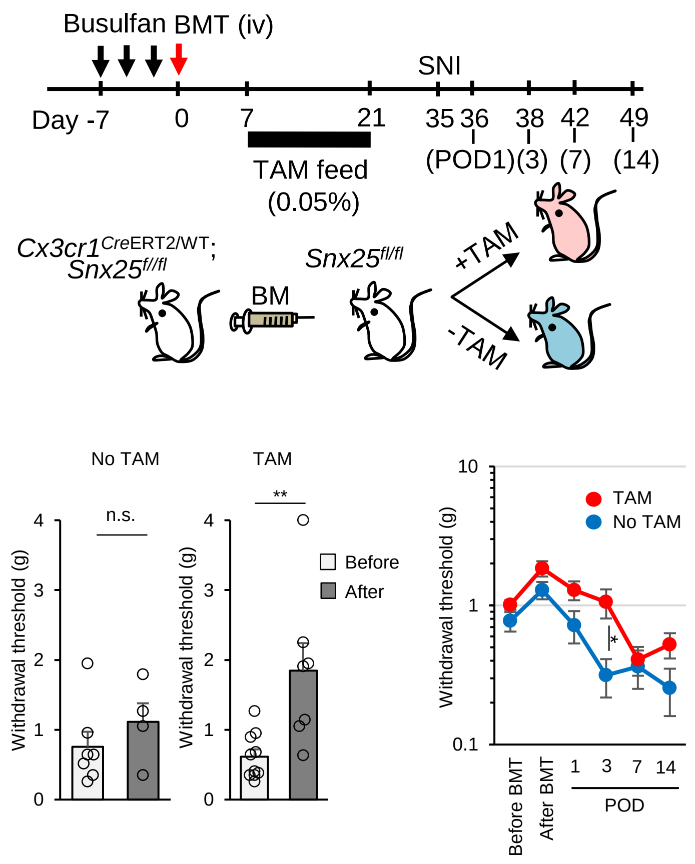

BM recipients were male 8-week-old mice. Mice were intraperitoneally injected with the chemotherapeutic agent busulfan (30 μg/g body weight; B2635, Sigma-Aldrich) in a 1:4 solution of dimethyl sulfoxide and PBS at 7, 5, and 3 days prior to bone marrow transfer. All mice were treated with antibiotics (trimethoprim (35039, Nacalai Tesque) and sulfamethoxazole (S7507, Sigma)) for 14 days after busulfan treatment. BM-derived cells were obtained from the femur and tibia of 5-week-old *Cx3cr1*$^{CreERT2/WT}$; *Snx25*$^{fl/fl}$ mice and resuspended in PBS with 2% FBS. BM-derived cells (1×10^6) were transferred to recipient mice by tail vein injection.

Supporting Data for Fig. 5

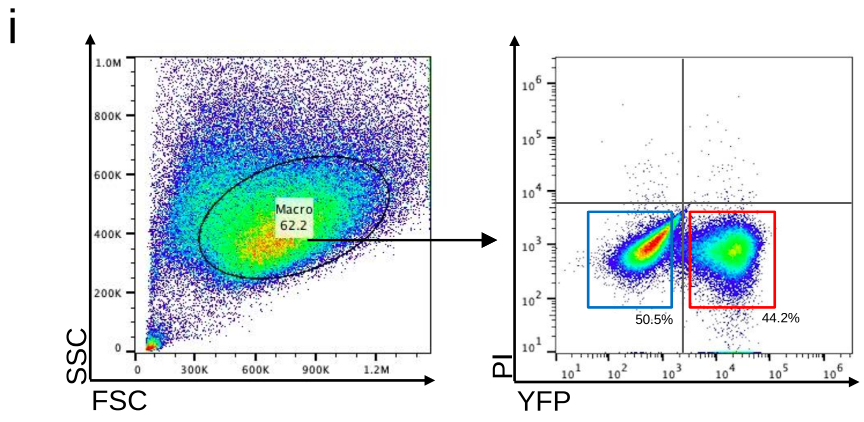

For analysis of BMDM generated from *Cx3cr1*$^{\mathit{Cre}\mathrm{ERT2/WT}}$; *Snx25*$^{fl/fl}$; Ai32/+ mice, BMDM were collected and stained with PI to exclude the dead cells from analysis. YFP positive or negative cells were isolated by the Cell Sorter SH800 (Sony).

Supporting Data for Fig. 7

b

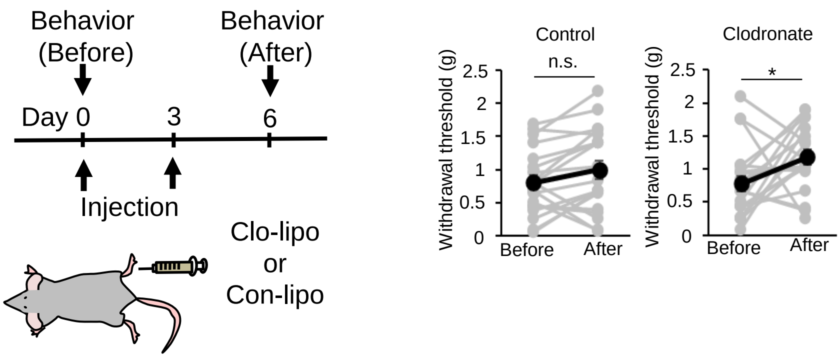

Twenty microliters of 10 mg/ml clodronate liposomes or control liposomes were subcutaneously injected into the right side of the hind paw skin on days 0 and 3.

Supporting Data for Fig. 7 (*Continued*)

e

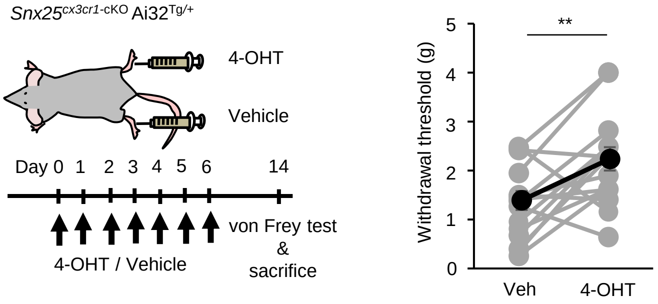

For depletion of SNX25 in dermal macrophages, we administered 4-OHT (40 ng/μl, 10 μl) by intradermal injection daily for seven days into *Cx3cr1*$^{Cre\mathrm{ERT2/WT}}$; *Snx25*$^{fl/fl}$; Ai32/+ mice. Vehicle was injected into the contralateral side of the same animal. At 8 days after the last injection, a von Frey test was performed.

Supporting Data for Fig. 8

j

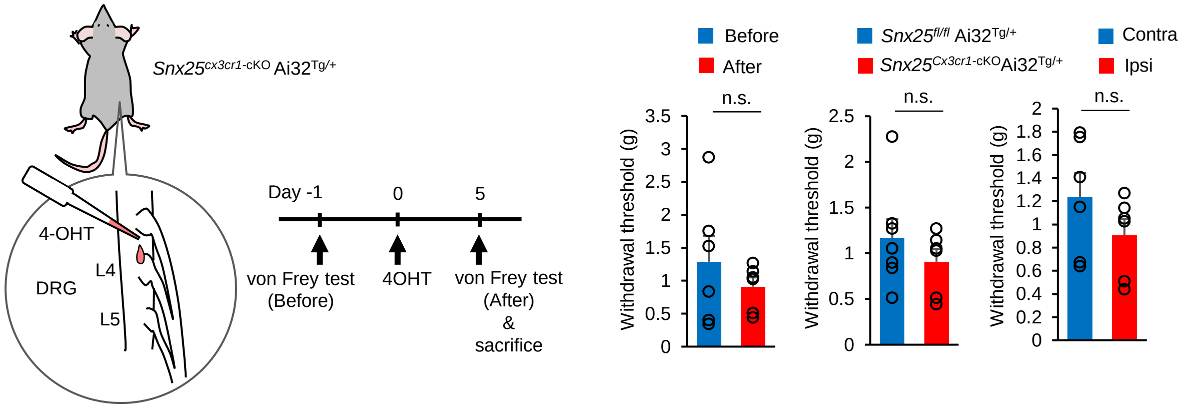

For depletion of SNX25 in the DRG, we injected 4-OHT (200 ng/µl, 20 µl) into exposed DRG (L4 and L5) of *Cx3cr1*$^{\mathit{Cre}\mathrm{ERT2/WT}}$; *Snx25*$^{fl/fl}$; Ai32/+ mice. At 5 days after administration, the von Frey test was performed.

Supporting Data for Extended Data Fig. 6

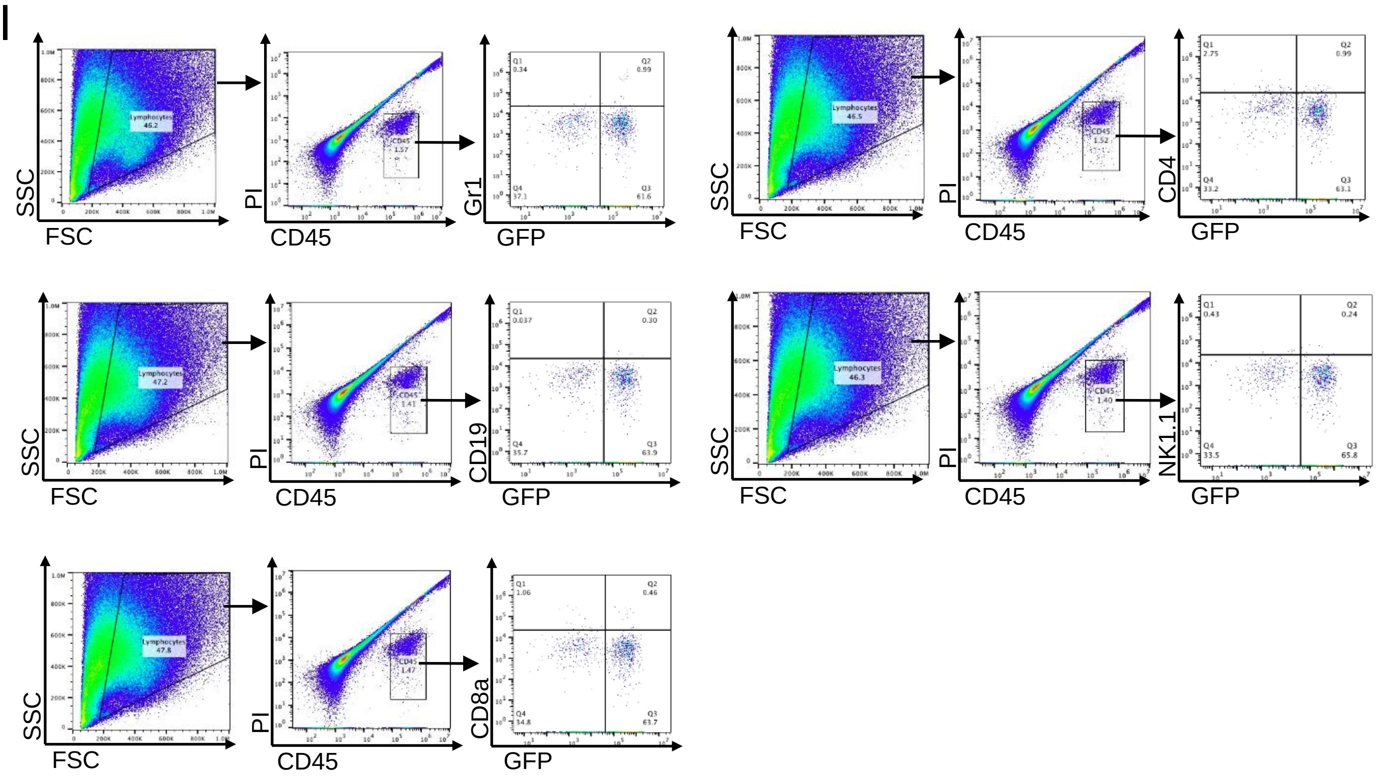

For the analysis of myeloid population in the skin, cells were obtained from hind paw skin using a Multi Tissue Dissociation Kit 1 (130-110-201, Miltenyi Biotec) with gentleMACS dissociator (Miltenyi Biotec) according to manufacturer's instruction. The hind paw skin were minced by razor blade, and then subjected to enzymatic digestion at 37 ºC, for 2 h in rotation. During the enzymatic digestion, cells were dispersed by the programs (h_tumor_01, h_tumor_02, and h_tumor_03) of gentleMACS. Debris was removed by a 70-µm cell strainer. In some experiments, cells were separated with 30/70% percoll (17-0891, GE Healthcare) by the centrifugation for 20 min at 400 *g*. The cells were stained with various combinations of mAbs. FcγII/III receptors were blocked by prior incubation with anti-CD16/32 antibody (1:100, 156604, BioLegend). The mAbs used in this study were PE–anti-CD45 (1:100, 103106, BioLegend), Biotin-anti-Ly6G/Ly6C (1:100, 108403, BioLegend), Biotin-anti-CD19 (1:100, 115504, BioLegend), Biotin-anti-CD8a (1:100, 100704, BioLegend), Biotin-anti-CD4 (1:100, 100404, BioLegend), Biotin-anti-NK1.1 (1:100, 108704, BioLegend) and second Ab APC-Streptavidin (1:200, 405207, BioLegend). To exclude dead cells from analysis, cells were stained with propidium iodide (PI, 421301, BioLegend). Cells were analyzed and sorted using Cell Sorter SH800S (Sony). Data were processed with FlowJo (v10, Tree Star).

Supporting Data for Extended Data Fig. 6 (*Continued*)

m

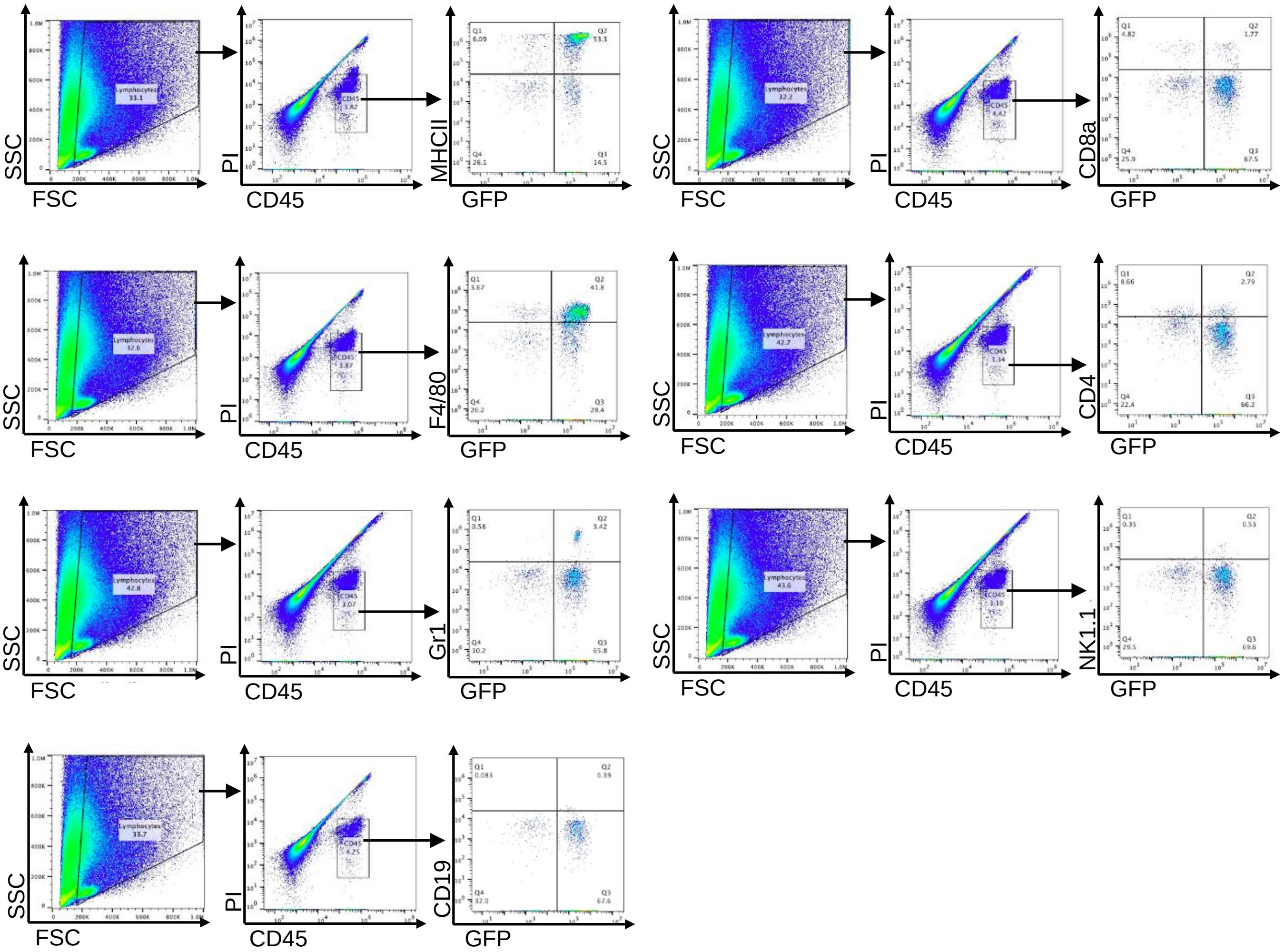

For the analysis of myeloid population in the skin, cells were obtained from back skin using a Multi Tissue Dissociation Kit 1 (130-110-201, Miltenyi Biotec) with gentleMACS dissociator (Miltenyi Biotec) according to manufacturer's instruction. The back skin dehaired using forceps were minced by razor blade, and then subjected to enzymatic digestion at 37 ^{o}C, for 2 h in rotation. During the enzymatic digestion, cells were dispersed by the programs (h_tumor_01, h_tumor_02, and h_tumor_03) of gentleMACS. Debris was removed by a 70-µm cell strainer. In some experiments, cells were separated with 30/70% percoll (17-0891, GE Healthcare) by the centrifugation for 20 min at 400 *g*. The cells were stained with various combinations of mAbs. FcγII/III receptors were blocked by prior incubation with anti-CD16/32 antibody (1:100, 156604, BioLegend). The mAbs used in this study were PE–anti-CD45 (1:100, 103106, BioLegend), Biotin-anti-MHCII (1:100, 107603, BioLegend), Biotin-anti-F4/80 (1:100, 123105, BioLegend), Biotin-anti- Ly6G/Ly6C (1:100, 108403, BioLegend), Biotin-anti-CD19 (1:100, 115504, BioLegend), Biotin-anti-CD8a (1:100, 100704, BioLegend), Biotin-anti-CD4 (1:100, 100404, BioLegend), Biotin-anti-NK1.1 (1:100, 108704, BioLegend) and second Ab APC-Streptavidin (1:200, 405207, BioLegend). To exclude dead cells from analysis, cells were stained with propidium iodide (PI, 421301, BioLegend). Cells were analyzed and sorted using Cell Sorter SH800S (Sony). Data were processed with FlowJo (v10, Tree Star).

Supporting Data for Extended Data Fig. 7

j

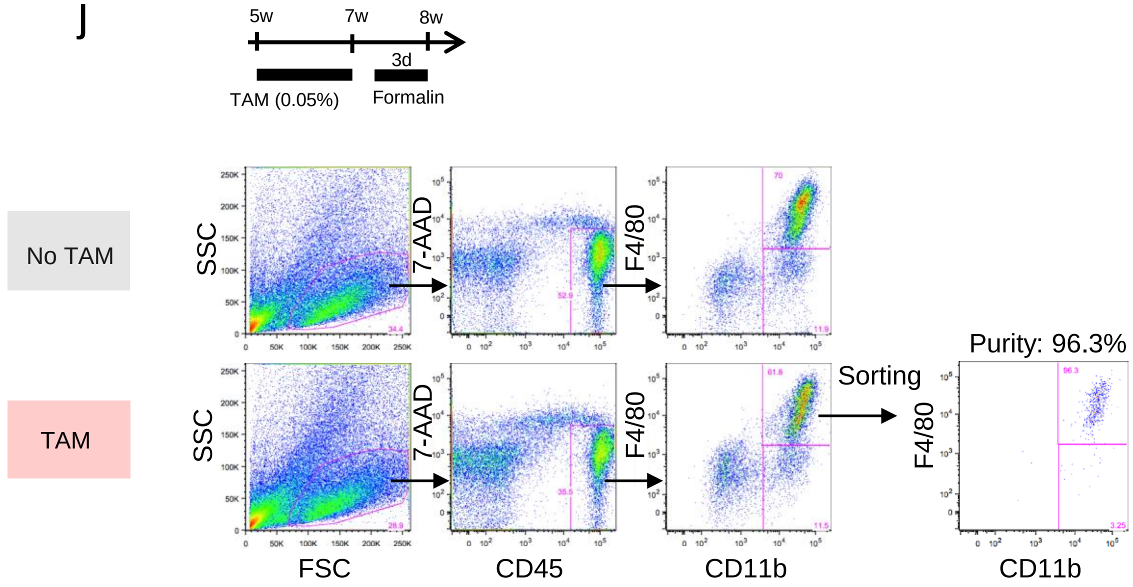

For the analysis of myeloid population in the skin, cells were obtained from hind paw skin using a Multi Tissue Dissociation Kit 1 (130-110-201, Miltenyi Biotec) with gentleMACS dissociator (Miltenyi Biotec) according to manufacturer's instruction. The hind paw skin were minced by razor blade, and then subjected to enzymatic digestion at 37 ^{o}C, for 2 h in rotation. During the enzymatic digestion, cells were dispersed by the programs (h_tumor_01, h_tumor_02, and h_tumor_03) of gentleMACS. Debris was removed by a 70-µm cell strainer. The cells were separated by the centrifugation for 20 min at 400 *g*. The cells were stained with various combinations of mAbs. FcγII/III receptors were blocked by prior incubation with anti-CD16/32 antibody (1:100, 156604, BioLegend). The mAbs used in this study were PE/Cyanine7 anti-CD45 (1:100, 103113, BioLegend), Fluor 488–anti-CD11b (1:100, 101219, BioLegend), Alexa Fluor 647-anti-F4/80 (1:100, 123121, Biolegend). To exclude dead cells from analysis, cells were stained with 7-AAD (559925, BD Biosciences). Cells were analyzed and sorted using FACSAria (BD biosciences). Data were processed with FlowJo (v10, Tree Star). Purity of CD45^{+}CD11b^{high}F4/80^{high} population from the hind paw skin was more than 96%.

Supporting Data for Extended Data Fig. 8

b

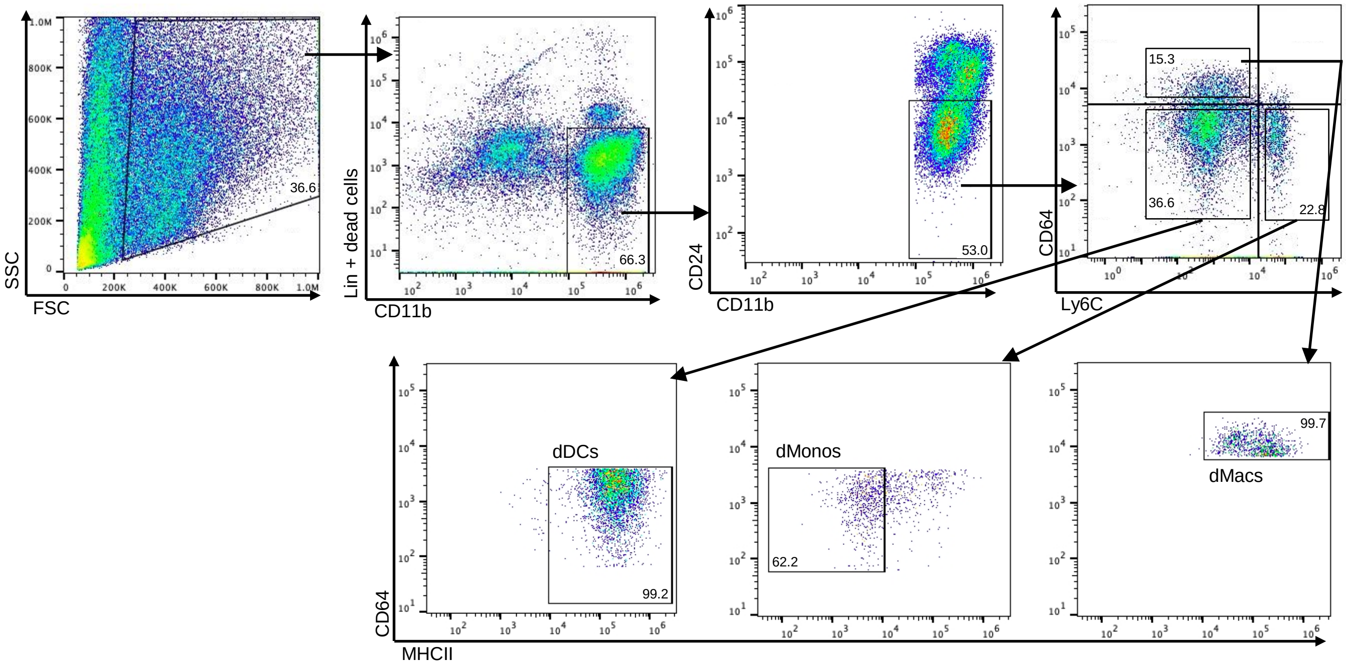

For the analysis of myeloid population in the skin, cells were obtained from back skin using a Multi Tissue Dissociation Kit 1 (130-110-201, Miltenyi Biotec) with gentleMACS dissociator (Miltenyi Biotec) according to manufacturer's instruction. The back skin dehaired using forceps were minced by razor blade, and then subjected to enzymatic digestion at 37 ^{o}C, for 2 h in rotation. During the enzymatic digestion, cells were dispersed by the programs (h_tumor_01, h_tumor_02, and h_tumor_03) of gentleMACS. Debris was removed by a 70-μm cell strainer. In some experiments, cells were separated with 30/70% percoll (17-0891, GE Healthcare) by the centrifugation for 20 min at 400 *g*. The cells were stained with various combinations of mAbs. FcγII/III receptors were blocked by prior incubation with anti-CD16/32 antibody (1:100, 156604, BioLegend). The mAbs used in this study were FITC–anti-MHCII (1:100, 107605, BioLegend), PE-anti-CD11b (1:100, 101207, BioLegend), APC anti-CD64 (1:25, 139306, BioLegend), APC/Cyanine7 Ly6C (1:100,128025, BioLegend), Brilliant violet 421 anti-CD24 (1:100, 101825, BioLegend), Biotin anti-CD3 (1:100, 100243, BioLegend), Biotin anti-TER119 (1:100, 116203, BioLegend), Biotin anti-Ly6G (1:100, 127603, BioLegend), and PerCP-Cyanine5.5 streptavidin (1:200, 405214, BioLegend). To exclude dead cells from analysis, cells were stained with Fixable Viability Stain 700 (564997, BD Biosciences). For isolation of myeloid populations from the back skin, sticky or dead cells, which none-specifically bind to microbeads and/or column, were removed by Basic MicroBeads (130-048-001, Miltenyi Biotec) with autoMACS separator, and then leukocytes were enriched by CD45 Microbeads (Miltenyi Biotec, 130-052-301). Dermal macrophages (CD64^{+}Ly6C^{-}MHCII^{+}), dermal monocytes (CD64^{-} Ly6C^{+} MHCII^{lo}), and dermal dendritic cells (CD64^{-}Ly6C^{-}MHCII^{+}) in CD11b^{+}Lin (CD3, CD19, CD34, Ly6G, NK1.1, and TER119) negative cells were isolated using a Cell Sorter SH800 (> 94% purity). Cells were analyzed and sorted using Cell Sorter SH800S (Sony). Data were processed with FlowJo (v10, Tree Star).
