# Supplementary material for: Dermal macrophages set pain sensitivity by modulating the amount of tissue NGF through an SNX25–Nrf2 pathway
Source: Nat Immunol. 2023 Jan 26;24(3):439–51. doi: 10.1038/s41590-022-01418-5 (PMC9977679; doi:10.1038/s41590-022-01418-5)
Supplement: Supplementary file 2 — Reporting Summary [file 41590_2022_1418_MOESM2_ESM.pdf]

Corresponding author(s): Tatsuhide Tanaka
Akio Wanaka

Last updated by author(s): Dec 15, 2022

Reporting Summary

Nature Portfolio wishes to improve the reproducibility of the work that we publish. This form provides structure for consistency and transparency in reporting. For further information on Nature Portfolio policies, see our Editorial Policies and the Editorial Policy Checklist.

Statistics

For all statistical analyses, confirm that the following items are present in the figure legend, table legend, main text, or Methods section.

n/a	Confirmed	
☐	☒	The exact sample size (*n*) for each experimental group/condition, given as a discrete number and unit of measurement
☐	☒	A statement on whether measurements were taken from distinct samples or whether the same sample was measured repeatedly
☐	☒	The statistical test(s) used AND whether they are one- or two-sided<br>*Only common tests should be described solely by name; describe more complex techniques in the Methods section.*
☐	☒	A description of all covariates tested
☐	☒	A description of any assumptions or corrections, such as tests of normality and adjustment for multiple comparisons
☐	☒	A full description of the statistical parameters including central tendency (e.g. means) or other basic estimates (e.g. regression coefficient) AND variation (e.g. standard deviation) or associated estimates of uncertainty (e.g. confidence intervals)
☐	☒	For null hypothesis testing, the test statistic (e.g. *F*, *t*, *r*) with confidence intervals, effect sizes, degrees of freedom and *P* value noted<br>*Give P values as exact values whenever suitable.*
☒	☐	For Bayesian analysis, information on the choice of priors and Markov chain Monte Carlo settings
☒	☐	For hierarchical and complex designs, identification of the appropriate level for tests and full reporting of outcomes
☒	☐	Estimates of effect sizes (e.g. Cohen's *d*, Pearson's *r*), indicating how they were calculated

Our web collection on statistics for biologists contains articles on many of the points above.

Software and code

Policy information about availability of computer code

Data collection	Images of micrographs were captured using a confocal laser scanning microscope (C2 ver4.10, Nikon).<br>Immunostained cell were collected using FACSAria by BD FACSDiva 4.1, or SONY cell sorter SH 800 series by software ver2.1 .
Data analysis	Adobe Photoshop CC 2018, ImageJ, Statcel-the Useful Addin Forms on Excel-4th ed, FlowJo software (V10), IBM SPSS (V23).

For manuscripts utilizing custom algorithms or software that are central to the research but not yet described in published literature, software must be made available to editors and reviewers. We strongly encourage code deposition in a community repository (e.g. GitHub). See the Nature Portfolio guidelines for submitting code & software for further information.

Data

Policy information about availability of data

All manuscripts must include a data availability statement. This statement should provide the following information, where applicable:

- Accession codes, unique identifiers, or web links for publicly available datasets
- A description of any restrictions on data availability
- For clinical datasets or third party data, please ensure that the statement adheres to our policy

All data supporting the findings of this study are found within the manuscript and its Supplementary information, and are available from the corresponding author upon reasonable request. Source data are provided with this paper.

Field-specific reporting

Please select the one below that is the best fit for your research. If you are not sure, read the appropriate sections before making your selection.

☒ Life sciences ☐ Behavioural & social sciences ☐ Ecological, evolutionary & environmental sciences

For a reference copy of the document with all sections, see nature.com/documents/nr-reporting-summary-flat.pdf

Life sciences study design

All studies must disclose on these points even when the disclosure is negative.

Sample size	No statistical methods were used to predetermine sample size, but our sample sizes are similar to those reported in previous publications. A minimum of three biologically independent samples were tested, and experiments performed in at least 2 independent instances (mostly 3) with similar results. Sample size was based on pilot experiments and previous experience with similar experiments in the laboratory. Data from individual experiments were pooled to achieve power.
Data exclusions	No data was excluded.
Replication	Behavioral experiments on mice were conducted with a sufficient number of animals (6 to 33) to take individual differences into account. Experiments were conducted at least five times. All experiments were performed in multiple independent experiments as indicated in the figure legends. All attempts for littermates were successfully performed by multiple investigators using independent litter of mice.
Randomization	Transgenic mice were predetermined by mouse genotype and therefore could not be randomized. Littermates were assigned into the control or knockout groups after genotyping. In all the behavioral tests, however, examiners were always blind to the genotypes of mice, the kinds of treatments, and the sides of hind paws that received injections. After the evaluation was done, the behavioral data were analyzed by a different researcher. C57BL/6J mice were randomly assigned to treatment and control groups. All mice were age-matched.
Blinding	Experimentors was blinded to the identify of mice being analyzed in behavioral tests. For experiments other than behavioral tests, experimentors were not blinded. Blinding was not possible as predominately one person was responsible for performing each experiment and carrying out data analysis. Blinding was not relevant because the quantification of signal intensity in Immunoblot or images, and analysis of gene expression level using thermal cycler were performed under the same conditions as in the control group, respectively.

Reporting for specific materials, systems and methods

We require information from authors about some types of materials, experimental systems and methods used in many studies. Here, indicate whether each material, system or method listed is relevant to your study. If you are not sure if a list item applies to your research, read the appropriate section before selecting a response.

Materials & experimental systems

n/a	Involved in the study	
☐	☒	Antibodies
☐	☒	Eukaryotic cell lines
☒	☐	Palaeontology and archaeology
☐	☒	Animals and other organisms
☒	☐	Human research participants
☒	☐	Clinical data
☒	☐	Dual use research of concern

Methods

n/a	Involved in the study	
☒	☐	ChIP-seq
☐	☒	Flow cytometry
☒	☐	MRI-based neuroimaging

Antibodies

Antibodies used

Mouse anti-NF200 (N0142, Sigma-Aldrich, clone N52) 1:1000, AB_477257
Mouse anti-PGP9.5 (ab8189, Abcam, clone 13C4 / I3C4) 1:500, AB_306343
Mouse anti-NeuN (MAB377, Millipore, clone A60) 1:150, AB_2298772
Mouse anti-GFAP (MAB360, Millipore, clone GA5) 1:500, AB_11212597
Rabbit anti-c-Fos (226003, Synaptic Systems) 1:10000, AB_2231974
Rabbit anti-TRPV1 (KM018, Trans Genic) 1:100, AB_1627247
Rabbit anti-TrkA (ab76291, Abcam) 1:150-1:1000, AB_1524514
Rabbit anti-Iba1 (019-19741, Wako) 1:500, AB_839504
Rabbit anti-F4/80 (28463-1-AP, Proteintech) 1:2000, AB_2881149
Rabbit anti-NGF (sc-548, Santa Cruz Biotechnology) 1:200-1:1000, AB_632011
Rabbit anti-GFP (A6455, Thermo Fisher Scientific) 1:5000, AB_221570
Rabbit anti-SNX25 (13294-1-AP, Proteintech) 1:500, AB_2192549
Rabbit anti-Nrf2 (sc-722, Santa Cruz Biotechnology) 1:500, AB_2108502
Rabbit anti-HO-1 (ADI-SPA-896, Enzo Life Sciences,) 1:500, AB_10614948

Rabbit anti-TGFbRI (sc-398, Santa Cruz Biotechnology) 1:200, AB_632493
Rabbit anti-GAPDH (ABS16, Merk Millipore) 1:2000, AB_10806772
Rat anti-MHCII (NBP1-43312, Novus Biologicals) 1:100, AB_10006677
Rat anti-F4/80 (NB600-404, Novus Biologicals) 1:500, AB_10003219
Rat anti-CD117 (MAB1356, R&D Systems) 1:100, AB_2131131
Rat anti-CCR2 (NBP1-48337, Novus Biologicals) 1:200, AB_10011101
Rat anti-GFP (04404-84, Nacalai Tesque) 1:5000, AB_10013361
Goat anti-CD206 (AF2535, R&D Systems) 1:500-1:1000, AB_2063012
Alexa Fluor 488–anti-CD11b (101219, BioLegend, clone, M1/70) 1:100, AB_493545
Alexa Fluor 647-anti-F4/80 (123121, Biolegend, clone, BM8) 1:100, AB_893480
APC anti-CD64 (139306, BioLegend, clone, X54-5/7.1) 1:25, AB_11219391
APC/Cyanine7 Ly6C (128025, BioLegend, clone HK1.4) 1:100, AB_10643867
Biotin anti-CD4 (100403, BioLegend, clone GK1.5) 1:100-1:200, AB_312688
Biotin anti-CD8a (100703, BioLegend, clone 53-6.7) 1:100-1:200, AB_312742
Biotin anti-Ly6G/Ly6C (108403, BioLegend, clone Gr1) 1:100-1:200, AB_313368
Biotin anti-NK1.1 (108703, BioLegend, clone PK136) 1:100-1:200, AB_313390
Biotin-anti-CD19 (115504, BioLegend, clone 6D5) 1:100, AB_313639
Biotin anti-CD19 (13-0193-81, eBioscience, clone 1D3) 1:100-1:200, AB_657657
Biotin anti-MHCII (107603, BioLegend, clone M5/114.15.2) 1:100, AB_313318
Biotin anti-F4/80 (123105, BioLegend, clone BM8) 1:100-1:500, AB_893499
Biotin anti-CD3 (100243, BioLegend, clone 17A2) 1:100, AB_2563946
Biotin anti-TER119 (116203, BioLegend, clone TER-119) 1:100, AB_313704
Biotin anti-Ly6G (127603, BioLegend, clone 1A8) 1:100, AB_1186105
FITC–anti-MHCII (107605, BioLegend, clone M5/114.15.2) 1:100, AB_313320
PE/Cyanine7 anti-CD45 (103113, BioLegend, clone 30-F11) 1:100, AB_312979
PE anti-CD11b (101207, BioLegend, clone M1/70) 1:100, AB_312790
PE anti-CD45 (103106, BioLegend, clone 30-F11) 1:100, AB_312971
BV421 anti-mouse CD45 (103131, BioLegend, clone 30-F11) 1:00, AB_10899570
Brilliant violet 421 anti-CD24 (101825, BioLegend, clone M1/69) 1:100, AB_10901159
PerCP-Cyanine5.5 streptavidin (405214, BioLegend) 1:200, AB_2716577
APC-Streptavidin (405207, BioLegend) 1:200
TruStain FcX™ PLUS (CD16/32 antibody, 156604, BioLegend, clone S17011E) 1:100, AB_2783138

Validation

All antibodies used are commercially available as described in the manuscript. We selected antibody clones that have been extensively used in the literature. We also titrated all antibodies prior to experiments.

Mouse anti-NF200 (N0142, Sigma-Aldrich, clone N52)
https://www.sigmaaldrich.com/deepweb/assets/sigmaaldrich/product/documents/355/551/n0142dat.pdf

Mouse anti-PGP9.5 (ab8189, Abcam, clone 13C4 / I3C4)
https://www.abcam.co.jp/pgp95-antibody-13c4--i3c4-ab8189.html

Mouse anti-NeuN (MAB377, Millipore, clone A60)
https://www.merckmillipore.com/JP/ja/product/Anti-NeuN-Antibody-clone-A60,MM_NF-MAB377

Mouse anti-GFAP (MAB360, Millipore, clone GA5)
https://www.merckmillipore.com/JP/ja/product/Anti-Glial-Fibrillary-Acidic-Protein-Antibody-clone-GA5,MM_NF-MAB360

Rabbit anti-c-Fos (226003, Synaptic Systems)
https://sysy.com/product/226003

Rabbit anti-TRPV1 (KM018, Trans Genic)
https://www.sceti.co.jp/images/upload/export/1013_KM018_p.pdf

Rabbit anti-TrkA (ab76291, Abcam)
https://www.abcam.co.jp/pan-trk-antibody-ep1058y-ab76291.html

Rabbit anti-Iba1 (019-19741, Wako)
https://labchem-wako.fujifilm.com/us/product/detail/W01W0101-1974.html

Rabbit anti-F4/80 (28463-1-AP, Proteintech)
https://www.ptglab.co.jp/products/F4-80-Antibody-28463-1-AP.htm

Rabbit anti-NGF (sc-548, Santa Cruz Biotechnology)
https://datasheets.scbt.com/sc-548.pdf

Rabbit anti-GFP (A6455, Thermo Fisher Scientific)
https://www.thermofisher.com/antibody/product/GFP-Antibody-Polyclonal/A-6455

Rabbit anti-SNX25 (13294-1-AP, Proteintech)
https://www.ptglab.co.jp/products/SNX25-Antibody-13294-1-AP.htm

Rabbit anti-Nrf2 (sc-722, Santa Cruz Biotechnology)
https://datasheets.scbt.com/sc-722.pdf

Rabbit anti-HO-1 (ADI-SPA-896, Enzo Life Sciences,)
https://www.enzolifesciences.com/ADI-SPA-896/ho-1-polyclonal-antibody/

Rabbit anti-TGFbRI (sc-398, Santa Cruz Biotechnology)
https://datasheets.scbt.com/sc-398.pdf

Rabbit anti-GAPDH (ABS16, Merk Millipore)
https://www.merckmillipore.com/JP/ja/product/Anti-GAPDH-Antibody,MM_NF-ABS16

Rat anti-MHCII (NBP1-43312, Novus Biologicals)
https://www.novusbio.com/products/mhc-class-ii-i-a-i-e-antibody-m5-114152_nbp1-43312

Rat anti-F4/80 (NB600-404, Novus Biologicals)
https://www.novusbio.com/products/f4-80-antibody-ci-a3-1_nb600-404

Rat anti-CD117 (MAB1356, R&D Systems)
https://www.rndsystems.com/products/mouse-cd117-c-kit-antibody-180627_mab1356

Rat anti-CCR2 (NBP1-48337, Novus Biologicals)
https://www.novusbio.com/products/ccr2-antibody_nbp1-48337

Rat anti-GFP (04404-84, Nacalai Tesque)
https://www.nacalai.co.jp/ss/ec2/EC-srchdetl.cfm?jump=EC-srchdetl&syohin=0440484&syubetsu=3

Goat anti-CD206 (AF2535, R&D Systems)
https://www.rndsystems.com/products/mouse-mmr-cd206-antibody_af2535

Alexa Fluor 488–anti-CD11b (101219, BioLegend, clone, M1/70)
https://www.biolegend.com/ja-jp/clone-search/alexa-fluor-488-anti-mouse-human-cd11b-antibody-2700

Alexa Fluor 647-anti-F4/80 (123121, Biolegend, clone, BM8)
https://www.biolegend.com/ja-jp/sean-tuckers-tests/alexa-fluor-647-anti-mouse-f4-80-antibody-4074

APC anti-CD64 (139306, BioLegend, clone, X54-5/7.1)
https://www.biolegend.com/ja-jp/clone-search/apc-anti-mouse-cd64-fcgammari-antibody-7874

APC/Cyanine7 Ly6C (128025, BioLegend, clone HK1.4)
https://www.biolegend.com/ja-jp/explore-new-products/apc-cyanine7-anti-mouse-ly-6c-antibody-6758?GroupID=BLG5853

Biotin anti-CD4 (100403, BioLegend, clone GK1.5)
https://www.biolegend.com/ja-jp/products/biotin-anti-mouse-cd4-antibody-247

Biotin anti-CD8a (100703, BioLegend, clone 53-6.7)
https://www.biolegend.com/ja-jp/productstab/biotin-anti-mouse-cd8a-antibody-152

Biotin anti-Ly6G/Ly6C (108403, BioLegend, clone Gr1)
https://www.biolegend.com/ja-jp/neuroscience-1/biotin-anti-mouse-ly-6g-ly-6c-gr-1-antibody-457?GroupID=BLG4876

Biotin anti-NK1.1 (108703, BioLegend, clone PK136)
https://www.biolegend.com/ja-jp/productstab/biotin-anti-mouse-nk-1-1-antibody-428

Biotin-anti-CD19 (115504, BioLegend, clone 6D5)
https://www.biolegend.com/ja-jp/products/biotin-anti-mouse-cd19-antibody-1527

Biotin anti-CD19 (13-0193-81, eBioscience, clone 1D3)
https://www.thermofisher.com/antibody/product/CD19-Antibody-clone-eBio1D3-1D3-Monoclonal/16-0193-81

Biotin anti-MHCII (107603, BioLegend, clone M5/114.15.2)
https://www.biolegend.com/fr-lu/products/biotin-anti-mouse-i-a-i-e-antibody-365?
pdf=true&displayInline=true&leftRightMargin=15&topBottomMargin=15&filename=Biotin%20anti-mouse%20I-A/I-E%
20Antibody.pdf

Biotin anti-F4/80 (123105, BioLegend, clone BM8)
https://www.biolegend.com/ja-jp/products/biotin-anti-mouse-f4-80-antibody-4066?GroupID=BLG5319

Biotin anti-CD3 (100243, BioLegend, clone 17A2)
https://production.biolegend.com/ja-jp/products/biotin-anti-mouse-cd3-antibody-10023

Biotin anti-TER119 (116203, BioLegend, clone TER-119)
https://www.biolegend.com/ja-jp/productstab/biotin-anti-mouse-ter-119-erythroid-cells-antibody-1864?GroupID=ImportedGROUP1

Biotin anti-Ly6G (127603, BioLegend, clone 1A8)
https://www.biolegend.com/ja-jp/products/biotin-anti-mouse-ly-6g-antibody-4772

FITC–anti-MHCII (107605, BioLegend, clone M5/114.15.2)
https://www.biolegend.com/ja-jp/clone-search/fitc-anti-mouse-i-a-i-e-antibody-366?GroupID=BLG11931

PE/Cyanine7 anti-CD45 (103113, BioLegend, clone 30-F11)
https://www.biolegend.com/ja-jp/products/pe-cyanine7-anti-mouse-cd45-antibody-1903

PE anti-CD11b (101207, BioLegend, clone M1/70)
https://www.biolegend.com/ja-jp/products/pe-anti-mouse-human-cd11b-antibody-349

PE anti-CD45 (103106, BioLegend, clone 30-F11)
https://www.biolegend.com/ja-jp/explore-new-products/pe-anti-mouse-cd45-antibody-100

BV421 anti-mouse CD45 (103131, BioLegend, clone 30-F11)
https://www.biolegend.com/ja-jp/explore-new-products/brilliant-violet-421-anti-mouse-cd45-antibody-7253

Brilliant violet 421 anti-CD24 (101825, BioLegend, clone M1/69)
https://www.biolegend.com/ja-jp/explore-new-products/brilliant-violet-421-anti-mouse-cd24-antibody-7323

PerCP-Cyanine5.5 streptavidin (405214, BioLegend)
https://www.biolegend.com/ja-jp/products/percp-cyanine5-5-streptavidin-4212
AAPC-Streptavidin (405207, BioLegend)
https://www.biolegend.com/ja-jp/explore-new-products/apc-streptavidin-1470

TruStain FcX™ PLUS (CD16/32 antibody, 156604, BioLegend, clone S17011E)
https://www.biolegend.com/en-us/punchout/punchout-products/trustain-fcx-plus-anti-mouse-cd16-32-antibody-17085?GroupID=GROUP20

Eukaryotic cell lines

Policy information about cell lines

Cell line source(s)	293T (RRID:CVCL_0063), RAW264.7 (RRID:CVCL_0493)
Authentication	293T cells (ECACC 12022001), RAW264.7 (ECACC 91062702)
Mycoplasma contamination	Cell-lines were not tested for mycoplasma contamination.
Commonly misidentified lines (See ICLAC register)	N/A

Animals and other organisms

Policy information about studies involving animals; ARRIVE guidelines recommended for reporting animal research

Laboratory animals

C57BL/6J, RRID:IMSR_JAX:000664

Mlc1Tgmice (B6; CBB6(129)-Tg(Mlc1-tTA)2Rhn), RBRC05450

Snx25 constitutive KO (Snx25+/-) mice (C57BL/6N-Atm1Brd Snx25tm1a(KOMP)Wtsi/NjuMmucd, strain number, T001400) , RRID:MMRRC_068035-UCD

CAG-Flpo mice (B6.Cg-Tg(CAG-FLPo)/1Osb), RBRC09982

Advillin-Cre mice (B6.Cg-Tg(Avil-Cre/ERT2)AJwo/J) (Jackson Laboratory, Stock No: 032027), RRID:IMSR_JAX:032027

Cx3cr1CreERT2 mice (B6.129P2(C)-Cx3cr1tm2.1(Cre/ERT2)Jung/J) (Jackson Laboratory, Stock No: 020940) , RRID:IMSR_JAX:020940

Ai39 mice, RCL-eNpHR3.0-EYFP, Jackson Laboratory, Stock No: 014539), RRID:IMSR_JAX:014539

Ai32 mice, RCL-ChR2(H134R)/EYFP, Jackson Laboratory, Stock No: 012569), RRID:IMSR_JAX:012569

GFP mice (C57BL/6-Tg (CAG-EGFP)), RBRC00267

All the protocols for the animal experiments were approved by the Animal Care Committee of Nara Medical University in accordance with the policies established in the NIH Guide for the Care and Use of Laboratory Animals. This study was also carried out in compliance with the ARRIVE guidelines (https://arriveguidelines.org/).

Wild animals	No wild animals were used in this study.
Field-collected samples	No field collected samples were used in this study.
Ethics oversight	All the protocols for the animal experiments were approved by the Animal Care Committee of Nara Medical University in accordance with the policies established in the NIH Guide for the Care and Use of Laboratory Animals. This study was also carried out in compliance with the ARRIVE guidelines (https://arriveguidelines.org/).

Note that full information on the approval of the study protocol must also be provided in the manuscript.

Flow Cytometry

Plots

Confirm that:

☒ The axis labels state the marker and fluorochrome used (e.g. CD4-FITC).

☒ The axis scales are clearly visible. Include numbers along axes only for bottom left plot of group (a 'group' is an analysis of identical markers).

☒ All plots are contour plots with outliers or pseudocolor plots.

☒ A numerical value for number of cells or percentage (with statistics) is provided.

Methodology

Sample preparation: Skin from mice were collected and dissociated using Multi Tissue Dissociation Kit 1 (Miltenyi Biotec, Germany).

Instrument: FACSAria (BD), Cell Sorter SH800 (Sony)

Software: Data was collected using BD FACSDiva Software and analyzed using FlowJo software (Tree Star).

Cell population abundance: Purity of sorted macrophages was more than 94%.

Gating strategy: For flow cytometry gating strategy for skin macrophages under inflammatory condition, cells isolated from hind paw skin were stained with CD11b, CD45 and F4/80. Living leukocytes were identified as 7-AAD negative CD45+ cells within medium forward scatter (FSC) and low side scatter (SSC) population. Macrophage compartment was identified as CD11b+F4/80+ cells within living leukocytes.

Dermal macrophages are defined by Fc-g receptor 1 (CD64) expression in lineage (CD3, CD19, Ly6G, NK1.1, TER119, and Langerhans cell marker CD24) negative CD45+ CD11b+ Ly6C- population and subdivided by the expression of MHCII. CD45 positive cells were isolated using magnetic beads from enzymatically digesting saline-perfused mouse skins, and then myeloid populations, including dermal macrophages (CD64+ Ly6C- MHCII+), dermal monocytes (CD64- Ly6C+ MHCII lo), and dermal dendritic cells (CD64- Ly6C- MHCII+) in the lineage- CD11b+ live cells were sorted.

For flow cytometry gating strategy for dermal myeloid cells including macrophages in mixed BM chimeric mice, dermal myeloid cells including macrophages were selectively collected from the skin of the mixed BM chimeric mice by FACS using FVS700, CD11b, CD45.1, CD45.2 and lineage (CD3, CD19, NK1.1, TER119, Ly6G) marker expression.

☒ Tick this box to confirm that a figure exemplifying the gating strategy is provided in the Supplementary Information.
